# Changes in Retinal Vasculature and Thickness after Small Incision Lenticule Extraction with Optical Coherence Tomography Angiography

**DOI:** 10.1155/2019/3693140

**Published:** 2019-05-20

**Authors:** Minjie Chen, Jinhui Dai, Lan Gong

**Affiliations:** ^1^Department of Ophthalmology, Eye and Ear, Nose, and Throat Hospital of Fudan University, 83 Fenyang Road, Shanghai 200031, China; ^2^Key Laboratory of Visual Impairment and Restoration of Shanghai, Fudan University, 83 Fenyang Road, Shanghai 200031, China; ^3^Key Myopia Laboratory of NHC, 83 Fenyang Road, Shanghai 200031, China; ^4^Key Laboratory of Myopia, Chinese Academy of Medical Science, 83 Fenyang Road, Shanghai 200031, China

## Abstract

**Purpose:**

To evaluate the changes in retinal vessel density and thickness after small incision lenticule extraction (SMILE) with optical coherence tomography angiography (OCTA) in myopic patients.

**Methods:**

In this prospective study, SMILE surgeries were done in 46 eyes of 24 patients with spherical equivalent (SE) more than −6.0 diopters (*D*). Retinal vessel density and thickness at the macula and optic nerve were recorded with OCTA before and 1 day, 2 weeks, and 1 month after surgery. Intraocular pressure (IOP), uncorrected distance visual acuity (UDVA), and refraction were taken at the same time.

**Results:**

The superficial retinal vessel density and deep foveal retinal vessel density 1 day after surgery were less than those before surgery; however, the changes at any timepoints were not statistically significant (*p*=0.2736 and *p*=0.1590, respectively). Both the superficial vessel density and deep vessel density at the parafoveal and perifoveal regions decreased significantly 1 day postoperatively (all *p* < 0.05) and then returned to the preoperative level at 2 weeks and stabilized thereafter. There were no significant changes in any of the 4 vessel densities in the area of peripapillary before and 1 day, 2 weeks, and 1 month after surgery (*p*=0.3345). No statistically significant differences between preoperative and postoperative retinal thickness were detected for the area of macula and optic nerve (all *p* > 0.5).

**Conclusions:**

The vessel density at the parafoveal and perifoveal regions decreased at 1 day after SMILE with no effect on the visual acuity and relieved within 2 weeks. Decreased ocular blood flow in response to the spike in IOP may account for such changes.

## 1. Introduction

Femtosecond laser-assisted corneal refractive surgeries hold major part in treating refractive errors nowadays. During these procedures, vacuum suction is inevitable. Previous studies have shown intraocular pressure (IOP) increased during suction-mediated application of the glass contact, which is used in flap creation and refractive cut during the femtosecond laser-assisted laser in situ keratomileusis (FS-LASIK) and femtosecond lenticule extraction (FLEx) and even femtosecond laser-assisted cataract surgery [1–3]. The sudden spike in IOP to levels exceeding 65 mmHg, which can damage the eye, has been observed during the traditional microkeratome flap creation of laser in situ keratomileusis (LASIK) [4, 5]. Femtosecond laser creation exerts less extreme IOP fluctuations but requires more procedural time than LASIK [3, 6, 7]. It is suggested that acute increases in IOP can induce ischemia-reperfusion injury, which may cause retinal ganglion cell death as well as damage to the optic nerve and retina [8, 9]. Though most researchers showed suction had no significant clinical effects on the macular and retinal nerve fiber layer (RNFL) thickness during FS-LASIK or FLEx [10–12], macular hemorrhage after FS-LASIK was reported in a patient with a moderate degree of myopia and no macular pathology [13]. Thus, the effect of sudden spike in IOP on the retina caused by suction during the femtosecond laser procedures is still a widespread concern among ophthalmologists.

Since 2011, the VisuMax femtosecond laser has been used to make the refractive cut in the small incision lenticule extraction (SMILE) procedure [14]. Studies have showed SMILE to be precise and accurate in treating refractive errors [15, 16]. The procedure of SMILE has two steps: creation of the lenticule with femtosecond laser and separation/extraction of the lenticule from the corneal stroma. However, whether the sudden spike in IOP and the lenticule separation during SMILE damage the retina remains unknown. Furthermore, Shoji et al. showed that a change in retinal vessel density could be detected before a change in ganglion cell complex (GCC) thickness occurs [17]. Therefore, the aim of this prospective study was to evaluate the changes in retinal vasculature and thickness after uncomplicated SMILE with optical coherence tomography angiography (OCTA) in myopic patients.

## 2. Methods

### 2.1. Participants

This study was approved by the Institutional Review Board of the Eye & ENT Hospital of Fudan University. All procedures adhered to the Declaration of Helsinki and were conducted in accordance with the approved research protocol. Informed consent was obtained from all participants before enrollment. Twenty-four patients (46 eyes) undergoing SMILE were enrolled in this prospective study from May 2018 to August 2018. Inclusion criteria were age 18 years or older, stable myopia for ≥2 years, corrected distance visual acuity of 20/25 or better, and spherical equivalent (SE) with subjective refraction more than −6.0 diopters (*D*). Exclusion criteria were a calculated postoperative residual stromal bed of <250 *μ*m and abnormal corneal topography. Eyes with media opacities that prevented good quality scans and any retinal or neurological disease were excluded (e.g., diabetics and evidence of glaucomatous optic nerve damage). The eyes with a past history of surgery, trauma, or inflammation were also excluded.

### 2.2. Surgical Techniques

A VisuMax (Carl Zeiss Meditec) femtosecond laser platform was used for all surgical procedures. Surgery was performed as described by a previous study and was done by the same experienced surgeon [18]. The suction time lasted for 24 seconds during the lenticule creation. Postoperatively, in addition to the regular topical antibiotics (ofloxacin ophthalmic solution 0.5%; Santen Pharmaceutical Co., Ltd.) and artificial tears (sodium hyaluronate eye drops 0.3%; Santen Pharmaceutical Co., Ltd.), topical steroids (fluorometholone 0.1%; Santen Pharmaceutical Co., Ltd.) were initially administered 6 times a day and tapered off over 20 days.

### 2.3. OCTA Data Acquisition and Processing

**Figure 1:**
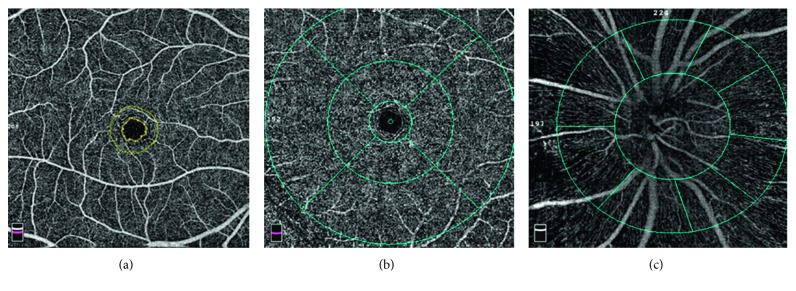
Optic coherence tomographic angiogram of the macular area and optic nerve from a 22-year-old male. (a) Foveal avascular zone (the area within the inner annulus). (b) Foveal area (the area within the inner circles) comprising the parafoveal area (the annulus between the inner and middle circles) and perifoveal area (the annulus between the middle and outer circles). The diameter of inner, middle, and outer circles is 1.0 mm, 3.0 mm, and 5.0 mm, respectively. (c) Peripapillary (the annulus between the inner and outer circles).

OCTA scans were obtained with a spectral-domain system (software version 2017.1.0.155; Optovue Inc., Fremont, CA, USA), a split-spectrum amplitude-decorrelation angiography algorithm to perform quantitative angiography of the retina. En face retinal angiograms were automatically created with projection from the internal limiting membrane to the retinal pigment epithelium. The software automatically calculated the perfused vessel density in the specific area of retina. Macular data were acquired over a 6.0 × 6.0 mm area. Retinal segmentation was automatically performed by the viewing software to generate en face projection images of the superficial retinal capillary plexus (SCP) and the deep retinal capillary plexus (DCP). The SCP en face OCTA image was segmented with an inner boundary 3 *μ*m below the internal limiting membrane and an outer boundary 15 *μ*m below the inner plexiform layer. The DCP en face OCTA image was set at 15 to 70 *μ*m beneath the inner plexiform layer. The foveal avascular zone (FAZ) was outlined and measured automatically by the software (Figure 1). The fovea is the 1.0 mm ring area at the center (Figure 1). The parafoveal region was defined as an annulus with an outer diameter of 3.0 mm and an inner diameter of 1.0 mm, and the perifoveal region was defined as an annulus with an outer diameter of 5.0 mm and an inner diameter of 3.0 mm (Figure 1). The optic nerve head data were covered by 4.5 × 4.5 mm OCTA scans. The peripapillary area was defined as a 700 mm wide elliptical annulus extending outward from the optic disc boundary (Figure 1). The software automatically calculated the perfused vessel density. Vessel density was defined as the percentage of the area occupied by vessels within the segmented area, as acquired by the provided RTVue-XR Avanti software.

Retinal thickness was obtained by the same OCT system at the same time as the retinal vasculature using the retina map mode. Retinal thickness referred to the mean thickness of that specific area. Macular thickness was calculated in the foveal, parafoveal, and perifoveal zones. The peripapillary RNFL was determined using the optic nerve head protocol. Measurements were automatically performed at four peripapillary quadrants: superior, inferior, nasal, and temporal according to the previous study [19]. We had a single observer to perform the OCTA examination who was blind to the study.

At the same time, heart rate (HR) and blood pressure (BP) were also measured and the mean arterial pressure was calculated as the diastolic blood pressure plus one-third of the difference between the diastolic and the systolic blood pressure [20, 21]. The ocular perfusion pressure was determined by subtracting the IOP from two-thirds of the mean arterial pressure. The signal strength index was used to control for image quality. Images with a signal strength index of less than 50 were excluded, and scans with movement or decentration artifacts were repeated. OCTA, uncorrected distance visual acuity (UDVA), HR, BP, and IOP (NIDEK TONOREF II; NIDEK Co., Ltd, Gamagori, Japan) and automated refraction (RK-8100, Topcon, Tokyo, Japan) measurements were recorded before and 1 day, 2 weeks, and 1 month after surgery. Subjective refraction was also measured before the surgery. To avoid the effects of diurnal variations, all measurements were obtained before the noon.

### 2.4. Statistical Analysis

Statistical analyses were performed using Stata 14.0 (Stata Corp., College Station, TX, USA). Quantitative data were expressed as the mean ± SD. Visual acuity was converted to LogMAR for data analysis. Analysis of variance (ANOVA) or the Kruskal–Wallis test was used to test for difference among different groups, and the Bonferroni test was used to identify which pairs of treated groups were significantly different. We also used multivariable linear regression model to detect parameters that could significantly predict the changes in vessel density after the surgery. Statistical significance was assumed at *p* < 0.05.

## 3. Results

Totally, 46 eyes (24 patients (20 men, 4 women)) were included in the final analysis. The demographic and clinical information of subjects is listed in Table 1. After the SMILE surgery, the LogMAR UDVA were −0.05 ± 0.06, −0.07 ± 0.04, and −0.08 ± 0.04 at 1 day, 2 weeks, and 1 month, which were significantly improved compared to the UDVA before surgery (*p*=0.0001). The difference in UDVA between 1 day and 2 weeks was insignificant (*p*=0.180). The similar result was found between 2 weeks and 1 month (*p*=0.527). However, the UDVA in 1 month postoperatively was significantly improved compared to that at 1 day (*p*=0.004). The IOP was 11.50 ± 2.09 mmHg, 11.95 ± 2.32 mmHg, and 11.38 ± 2.33 mmHg at 1 day, 2 weeks, and 1 month postoperatively, respectively (all *p* > 0.5). The mean BP and HR remained unchanged during all visits (all *p* > 0.5). A significant improvement was seen in SE at all follow-ups in contrast to the value preoperatively (*p*=0.0001), whereas the SE remained unchanged from 1 day to 1 month (all *p* > 0.5).

**Figure 2:**
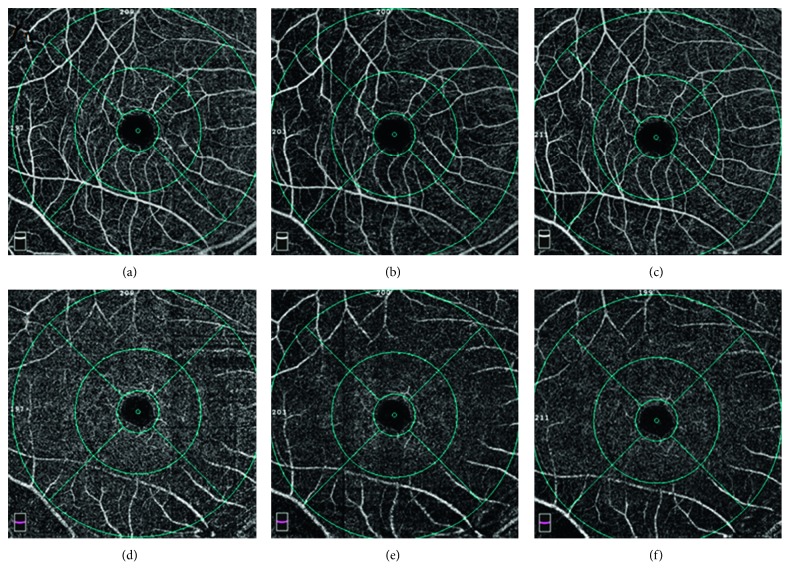
Optic coherence tomographic angiogram of the macular area from a 24-year-old female. The superficial vessel density at the fovea, parafovea, and perifovea was 16.3%, 55.4%, and 51.9% before surgery (a). The superficial vessel density at the fovea, parafovea, and perifovea was 12.2%, 43.5%, and 41.9% at 1 day postoperatively (b). The superficial vessel density at the fovea, parafovea, and perifovea was 14%, 51.3%, and 47.9% at 2 weeks postoperatively (c). The deep vessel density at the fovea, parafovea, and perifovea was 28%, 56.5%, and 51.3% preoperatively (d). The deep vessel density at the fovea, parafovea, and perifovea was 26%, 54%, and 42.3% at 1 day postoperatively (e). The deep vessel density at the fovea, parafovea, and perifovea was 26.4%, 54.1%, and 45.7% at 2 weeks postoperatively (f).

There were no significant changes in any of the 4 vessel densities in the area of peripapillary before and after surgery (*p*=0.3345) (Table 2). The superficial and deep foveal retinal vessel density at 1 day after surgery were less than those before surgery; however, the changes were not statistically significant (*p*=0.2736 and *p*=0.1590, respectively). The superficial retinal vessel density of the parafoveal and perifoveal regions decreased significantly at 1 day after surgery (*p*=0.006 and *p* < 0.001, respectively). At 2 weeks after surgery, there was a recovery in vessel density to the preoperative level at the parafoveal and perifoveal regions, respectively (*p*=0.011 and *p*=0.001, respectively) (Figure 2). There were no significant differences among the 3 superficial retinal vessel densities in the parafoveal and perifoveal regions before and 2 weeks and 1 month after surgery (all *p* > 0.5) (Table 2). The similar changes were seen in the deep retinal vessel density of the parafoveal and perifoveal regions (Table 2). The FAZ was 0.30 ± 0.10 mm^2^ before surgery and increased to be 0.50 ± 1.32 mm^2^ at 1 day after surgery. Then, the FAZ returned to the preoperative level of 0.30 ± 0.11 mm^2^ at 2 weeks and 0.30 ± 0.10 mm^2^ at 1 month after the surgery. Nevertheless, the differences in FAZ at the four timepoints were not statistically significant (*p*=0.9221).

Furthermore, we studied the parameters that could predict the changes in retinal vessel density of the parafoveal and perifoveal regions 1 day after the surgery. However, neither the ablation depth (AD) nor the preoperative SE was significantly correlated with the changes in retinal vessel density of the parafoveal and perifoveal regions 1 day after the surgery (all *p* > 0.5). Moreover, the changes in retinal vessel density of the parafoveal and perifoveal regions 1 day after the surgery had no significant influence on the improvement of UDVA at 1 day (*r* = 0.1137, *p*=0.4519; and *r* = 0.0225, *p*=0.8820, respectively). The preoperative IOP value and axial length were not correlated with the changes in retinal vessel density of the parafoveal and perifoveal regions 1 day after the surgery (all *p* > 0.5).

The foveal, parafoveal, and perifoveal retinal thicknesses at 3 timepoints postoperatively were not statistically different from those before the SMILE procedure (Table 3; all *p* > 0.5). Similarly, no statistically significant differences between preoperative and postoperative RNFL thickness values were detected for any of the examined sectors and the average peripapillary area (Table 3).

## 4. Discussion

OCTA is an imaging technology in vivo that provides detailed morphologic and quantitative microvascular information. A study with OCTA reported a reduced retinal vessel density in eyes with high myopia [22]. However, others found no significant differences among patients with mild myopia, moderate myopia, and high myopia [23–25]. It was speculated that pathologic changes in high myopia affect the macular vascular density rather than the SE. Nevertheless, in the present study, a significant decrease in vessel density at the parafovea and perifovea regions was found at 1 day after SMILE surgery in non-high myopia.

In eyes with high myopia, excessive axial elongation of the eyeball is usually accompanied with mechanical stretching of the retina, choroid, and sclera, leading to straightening and narrowing of the vessels [26, 27]. It should not be applied to the subjects of the non-high myopia in our study. Although the decrease in vessel density was reversible and completely recovered within 2 weeks in our study, it was proved for the first time that the procedure of SMILE had influence on the retinal vasculature at the macular area. We assumed the sudden spike in IOP during the SMILE procedure accounts for the changes. It was supported by Grunwald et al.'s study with a significantly faster leukocyte speed in the macula responding to a drop in IOP [28] and Weigert et al.' report with decreased fundus pulsation amplitude in response to an increase in IOP [29]. The refractive cut by femtosecond laser in SMILE was almost identical with the process in FLEx. It was reported that the increase in IOP during FLEx was similar to that in FS-LASIK, except for the duration of elevated IOP in FLEx was twice than that in FS-LASIK [2]. In another word, in addition to the elevation of the IOP (about 30 mmHg), the longer duration with the suction in SMILE may also cause a decrease in the ocular blood flow. Though the IOP fell steeply down in the suction off stage, the IOP was monitored to be still elevated during the process of lenticule separation/extraction in the animal model [30]. The long-lasting duration of elevated IOP in the eyes with SMILE surgery may lead to decreased fundus pulsation amplitude [29]. Consequently, the decreases in vessel density were observed by OCTA at 1 day postoperatively in the current study. However, the changes in vessel density at the parafoveal and perifoveal regions were transient and reversible as evidenced by the complete recovery at 2 weeks and 1 month after surgery. Moreover, the UDVA was significantly improved and remained stable at 3 postoperative timepoints in spite of the decrease in macular retinal vessel density. Thus, the temporary changes in retinal vessel density did not have impact on the recovery of visual acuity after the SMILE.

Though a significant decrease in macular vessel density was found 1 day after surgery in our study, the changes in retinal thickness and RNFL thickness were not detectable. This finding is in agreement with Shoji et al.'s indication that the change in retinal vessel density could be detected before a change in GCC thickness occurs [17]. It is also consistent with a previous study in which suction had no significant clinical effects on the macular thickness and RNFL thickness during FLEx or FS-LASIK [10]. However, it was demonstrated in another study that the average foveal and parafoveal retinal thicknesses were significantly thicker 1 day after surgery [11]. And the changes were reversible and recovered within 1 week without effect on the visual acuity [11]. We speculated the disparity of age accounts for the difference as the age is 9.68 ± 2.56 years in previous study compared to 21.00 ± 2.47 years in ours. It seemed that the SMILE procedure had no impact on the optic nerve as there were no changes either in the RNFL thickness or in the vessel density. Patients were instructed to stare at a specific light source for gaze fixation during the procedure. Thus, we speculated that the sudden spike in IOP may directly deliver to the macular area rather than to the optic nerve.

There are some limitations in our study. First, the small sample and the involvement of both right and left eyes are big issues. Though changes were seen in foveal vessel density and FAZ at 1 day after the surgery, neither of the differences were significant. The relatively small sample may account for the insignificance. Then, the interval between the 1 day and 2 weeks may not be appropriate. Because it has been reported in previous study that the foveal center retinal thickness after FS-LASIK was always relieved within 1 week [11]. Consequently, the duration of the alterations in the retinal vasculature after SMILE cannot be concluded in the present study. Finally, there were only 4 females in present study, and the proportion (20 males/4 females) may be not appropriate. However, several studies indicated gender did not significantly influence perfused vessel density in either the parafoveal or peripapillary region [20, 29].

## 5. Conclusion

The vessel density at the parafoveal and perifoveal regions decreased significantly at 1 day after the SMILE procedure, and these changes were not accompanied with the changes in macular thickness and with no effect on the visual acuity. Moreover, such decrease in vessel density was completely relieved at 2 weeks postoperatively. The SMILE procedure had no significant influence on the RNFL thickness and vessel density of the optic nerve. Decreased ocular blood flow in response to the spike in IOP may account for such changes.

## Figures and Tables

**Table 1:** Demographic data.

Characteristics	Mean ± SD
Age (years)	21.00 ± 2.47
SE (diopters)	−4.43 ± 1.80
Axial length (mm)	25.77 ± 0.99
UDVA	0.61 ± 0.33
IOP (mmHg)	15.98 ± 2.37
CCT (*μ*m)	538.61 ± 24.38
AD (*μ*m)	98.17 ± 27.37

SE, spherical equivalent; UDVA, uncorrected distance visual acuity; IOP, intraocular pressure; CCT, central corneal thickness; AD, ablation depth; SD, standard deviation.

**Table 2:** Vessel density (%) before and after SMILE with OCTA.

	Follow-up visits				
	Before surgery	1 day after surgery	2 weeks after surgery	1 month after surgery	*p*
Peripapillary	51.07 ± 3.22	51.84 ± 2.80	52.29 ± 3.90	51.42 ± 3.24	0.3345

*SCP*					
Fovea	21.19 ± 7.17	18.65 ± 7.14	20.50 ± 7.43	20.95 ± 7.31	0.2736
Parafovea	52.91 ± 8.13	48.97 ± 4.66	52.75 ± 4.94	53.25 ± 3.98	0.0001
Perifovea	51.77 ± 3.40	48.45 ± 3.53	51.19 ± 3.57	51.25 ± 3.27	0.0001

*DCP*					
Fovea	36.49 ± 9.35	33.26 ± 7.85	36.23 ± 7.94	36.33 ± 7.75	0.1590
Parafovea	56.02 ± 4.64	51.13 ± 4.45	55.41 ± 4.33	55.06 ± 4.39	<0.0001
Perifovea	52.34 ± 6.89	44.57 ± 5.74	51.10 ± 6.66	50.84 ± 7.17	0.0001

SMILE, small incision lenticule extraction; OCTA, optical coherence tomography angiography; SCP, superficial retinal capillary plexus; DCP, deep retinal capillary plexus.

**Table 3:** Peripapillary RNFL and macular thickness values before and after surgery (*μ*m).

	Before surgery	1 day after surgery	2 weeks after surgery	1 month after surgery	*p*
*Peripapillary RNFL*					
Mean	115.50 ± 12.63	117.33 ± 12.99	119.53 ± 16.44	116.75 ± 12.53	0.5893
Superior	137.04 ± 16.47	137.96 ± 17.60	140.16 ± 15.34	139.14 ± 15.81	0.7312
Inferior	139.87 ± 16.08	142.68 ± 18.55	141.76 ± 15.54	141.53 ± 15.70	0.9236
Temporal	89.41 ± 15.90	89.75 ± 12.38	87.06 ± 12.81	87.18 ± 14.07	0.7014
Nasal	99.52 ± 22.04	104.67 ± 30.64	109.56 ± 41.97	102.86 ± 22.44	0.3786

*Macular thickness*					
Foveal	244.46 ± 17.47	244.22 ± 17.51	245.07 ± 18.17	243.59 ± 17.28	0.9902
Parafoveal	321.15 ± 11.07	320.57 ± 10.74	320.05 ± 11.71	319.86 ± 11.83	0.9047
Perifoveal	279.78 ± 9.06	279.02 ± 9.17	279.14 ± 10.34	279.05 ± 10.14	0.9241

RNFL, retinal nerve fiber layer.

## Data Availability

The data used to support the findings of this study are available from the corresponding author upon request.
